# Music Communicates Affects, Not Basic Emotions – A Constructionist Account of Attribution of Emotional Meanings to Music

**DOI:** 10.3389/fpsyg.2018.00215

**Published:** 2018-02-28

**Authors:** Julian Cespedes-Guevara, Tuomas Eerola

**Affiliations:** ^1^Departamento de Estudios Psicológicos, ICESI University, Cali, Colombia; ^2^Department of Music, Durham University, Durham, United Kingdom

**Keywords:** music, basic emotions, dimensions, constructionism, perception of musical emotions, emotional expression in speech

## Abstract

Basic Emotion theory has had a tremendous influence on the affective sciences, including music psychology, where most researchers have assumed that music expressivity is constrained to a limited set of basic emotions. Several scholars suggested that these constrains to musical expressivity are explained by the existence of a shared acoustic code to the expression of emotions in music and speech prosody. In this article we advocate for a shift from this focus on basic emotions to a constructionist account. This approach proposes that the phenomenon of perception of emotions in music arises from the interaction of music’s ability to express core affects and the influence of top-down and contextual information in the listener’s mind. We start by reviewing the problems with the concept of Basic Emotions, and the inconsistent evidence that supports it. We also demonstrate how decades of developmental and cross-cultural research on music and emotional speech have failed to produce convincing findings to conclude that music expressivity is built upon a set of biologically pre-determined basic emotions. We then examine the cue-emotion consistencies between music and speech, and show how they support a parsimonious explanation, where musical expressivity is grounded on two dimensions of core affect (arousal and valence). Next, we explain how the fact that listeners reliably identify basic emotions in music does not arise from the existence of categorical boundaries in the stimuli, but from processes that facilitate categorical perception, such as using stereotyped stimuli and close-ended response formats, psychological processes of construction of mental prototypes, and contextual information. Finally, we outline our proposal of a constructionist account of perception of emotions in music, and spell out the ways in which this approach is able to make solve past conflicting findings. We conclude by providing explicit pointers about the methodological choices that will be vital to move beyond the popular Basic Emotion paradigm and start untangling the emergence of emotional experiences with music in the actual contexts in which they occur.

## Introduction

One of music’s most pervasive features is its power to represent or express meanings. All over the world people use music to symbolize a wide variety of meanings that range from national identities, and religious and political ideologies, to intimate personal connotations (Schubert, 2009; Clayton, 2016). Among this variety of meanings, the ability of music to represent or express emotions stands out as one of the main reasons why music is omnipresent in commercial environments, television, cinema, and the internet (North and Hargreaves, 2008), and is one of the main motivations why people devote so much time, energy and money to it (Kawase and Obata, 2016; Lamont et al., 2016). In developed societies, music has become one of the most important strategies for creating, enhancing, and modulating emotions (Thayer et al., 1994; Juslin and Laukka, 2004; Batt-Rawden and DeNora, 2005; Saarikallio, 2011; van Goethem and Sloboda, 2011).

During the last two decades, the emotional power of music has received increasing interest from psychology researchers, who have focused on two phenomena: the ability of music to arouse emotions, and its ability to express them. While this second line of research has amassed an impressive amount of evidence about how particular musical structures are related with listeners’ perception of emotion (Gabrielsson, 2009; Juslin and Timmers, 2010), it has not captured the richness and variety of emotional and non-emotional meanings that music represents in everyday contexts. On the contrary, influenced by the Basic Emotions theoretical framework (Tomkins, 1962; Izard, 1977; Ekman, 1992; Panksepp, 2000), most researchers in music psychology have restricted their investigation to music’s ability to express a limited set of so-called “basic emotions,” usually Happiness, Sadness, Fear, and Anger, and sometimes tenderness or love too (Krumhansl, 1997; Kallinen, 2005; Mohn et al., 2010; Juslin, 2013; Koelsch, 2014; see Eerola and Vuoskoski, 2013 for a review). Other researchers, influenced by Russell’s (1980) circumplex model, have investigated the phenomenon of musical expressivity in terms of more basic dimensions of affect (usually arousal and valence, e.g., Schubert, 1999; Gomez and Danuser, 2007; Egermann et al., 2009), while others have used other dimensions (such as tension and energy, e.g., Illie and Thompson, 2006, 2011) or *ad hoc* lists of emotional adjectives (e.g., Wedin, 1972; Giomo, 1993; Leman et al., 2005).

Most research about musical expressivity has been carried without discussing why music expressivity should be organized around discrete, basic emotions, or around more fundamental affective dimensions (Eerola and Vuoskoski, 2013). Two important exceptions are a study by Eerola and Vuoskoski (2011) that compared perceived emotions in music using the discrete emotion model, and the dimensional model of affect, and concluded that although there is a high correspondence between both models, the dimensional model is better suited to rate musical experts with ambiguous emotional expressivity. The second exception is Flaig and Large’s (2014) theory of dynamic communication of core affect, according to which, musically induced neural resonance communicates affect by modulating the listener’s arousal (via variations in tempo and intensity), and valence (via violation of musical expectations). In this paper, we focus on reviewing the evidence for the view that music expresses basic emotions, and like Flaig and Large (2014), we propose that adopting a dimensional model is a more fruitful framework to musical expressivity. However, unlike their theory, our theory does not deal with the underlying neural mechanisms that produce the modulations in listener’s arousal and valence.

Among those researchers who have studied musical expressivity in terms of discrete emotions, Juslin and colleagues have been the strongest advocates for the view that perception of emotions in music is based on the resemblance between vocal and musical expression of a set of basic emotions (Juslin, 1997, 2013; Juslin and Laukka, 2001, 2003; Lindström et al., 2003; Juslin and Timmers, 2010). Drawing from theories such as Ekman’s (1992) and Panksepp’s (2000), Juslin and colleagues theorize that there is a shared acoustic code to the expression of emotions in music and speech prosody, and that this code is organized into discrete categories, called “basic emotions.” In this perspective, basic emotions are considered innate and universal affect programs, which evolved through phylogenesis to serve important survival functions. Several empirical predictions are derived from this view of emotional expressivity: facial and vocal expressions of basic emotions (and therefore musical expressions of basic emotions too) are more readily perceived than expressions of non-basic emotions; basic emotions are expressed and perceived equally across cultures; appear early in development (Izard and Malatesta, 1987); have distinct brain substrates (Panksepp, 2000); are associated with distinct patterns of physiological activation (Ekman et al., 1983); and form the basis for other, non-basic emotions (Plutchik, 1980; Izard, 1992). Additionally, vocal and facial emotional expressions can also be identified in other species (Geen, 1992).

This Basic Emotions approach to musical expressivity underlies Juslin’s models of musical meaning: their theory of musical expressivity, and their model of musical communication.

Juslin’s theory of musical expressivity proposes that perception of musical emotions is based on three “layers” of coding of musical expression, which communicate basic emotions, tension, and arbitrary associations, respectively (Juslin, 2013). His second approach to musical meaning consists of a “lens model” of musical communication (Juslin, 1997, 2003; Juslin and Lindström, 2010). According to this model, senders (i.e., music performers or people talking emotionally) use a number of probabilistic and partly redundant acoustic cues to encode their emotional message. These cues leave traces in the acoustic object which can be subsequently detected by receivers (i.e., music listeners or conversation partners), who use them to decode and identify the intended emotion. Each cue in isolation is not a perfect indicator of the expressed emotion, and therefore the more cues are present in the acoustic object, and the more cues are used by decoders, the more likely it is that accurate communication takes place. Additionally, because some of the cues are partly redundant (i.e., they are associated with the same expressive intention), there are several cue combinations that can lead to successful communication.

The aim of this paper is to challenge the view that musical expressivity is organized around a set of discrete, basic emotions, and to propose an alternative, constructionist account of how the phenomenon of perceiving (or rather attributing) emotions expressed by music arises from our ability to detect the variations of arousal and valence specified by the musical sounds, and processes of categorization that relate those variations with contextual, situational, and personal cues. This interaction between perception of affect and categorization produces the experience of perceiving that a piece of music expresses emotions as if they were somehow “within” the musical sounds. In the first section of the paper we criticize the concept of basic emotions. Subsequently, we review the problematic evidence that supports the existence of shared acoustic code to the expression of basic emotions in vocalizations and music. Finally, we propose a constructionist account of the perception of musical emotions that overcomes the problems derived from applying the concept of Basic Emotions for musical expressions of emotion, and we discuss its implications for future research.

## The Problems With the Concept of Basic Emotions

The scholars who defend the concept of Basic Emotions conceive them as biologically primitive (i.e., supported by hardwired, discrete biological subsystems) and/or as psychologically primitive (i.e., as having elementary eliciting conditions, and forming the basis for other emotions) (Ortony and Turner, 1990; Scarantino and Griffiths, 2011). The biological primitiveness assumption is contradicted by findings that the same biological subsystems serve emotional and non-emotional psychological processes, and that even structures traditionally associated with discrete emotions (e.g., amygdala and fear), are involved in several emotions such as anger, happiness, and sadness (Lindquist et al., 2012; Raz et al., 2016). The psychological primitiveness assumption, in turn, is challenged by the consideration that several emotions traditionally considered as “basic,” share more elementary components. For instance, Anger, Sadness, and Disgust share a component of displeasure; and both Anger and Fear involve an evaluation of a situation as obstructing the realization of the individual’s goals (Ortony and Turner, 1990; Scherer, 2009).

A second set of problems with the Basic Emotion construct is that those who defend it do not agree on which emotions should be considered “basic.” Every author who proposes the existence of basic emotions has submitted a different list, ranging from two categories (Weiner and Graham, 1984) to ten (Izard, 1977). For instance, whereas Panksepp (2007) identifies seven “basic emotional responses” (Seeking, Rage, Fear, Lust, Care, Panic, and Play), Ekman and Cordaro (2011) propose somewhat different seven categories (Anger, Fear, Surprise, Sadness, Disgust, Contempt, and Happiness). Moreover, “love” or “tenderness,” an emotion included by Juslin (2013) in the list of basic emotions that vocalizations and music are able to express only appears in 4 out of the 14 theories reviewed by Ortony and Turner (1990). This figure increases to five theories if we consider Panksepp’s (2007) “care” category as equivalent.

In a paper dedicated to presenting his theory of how music expresses basic emotions, Juslin (2013, p. 6) argues that these disagreements do not constitute a problem, because the concept of basic emotions has heuristic value for the researchers who have adopted it, and because there is greater agreement about which emotions should be considered basic, than about how emotions should be defined in general. In our view, these arguments do not solve the problem. First, the fact that affective science has a problem agreeing on a definition of emotion is very serious, but probably not as insurmountable as Juslin makes it appear to be, as demonstrated by the similarities between several recent consensual definitions such as Scherer’s (2005), Frijda’s (2008), and Juslin and Sloboda (2010). Second, the existence of that lack of consensus does not make the lack of agreement among Basic Emotion theorists less serious. Third, even though it is true that several research programs have used the basic emotions concept in a heuristic manner, the fact that their lists and definitions do not match completely has made it difficult to accumulate the evidence into a single coherent conceptual framework. For instance, since anxiety, stress, distress, fear, and terror are similar but not identical states and concepts, the conclusions of research into these affective states are not necessarily consistent (c.f. Kreibig, 2010, p. 410). Finally, this narrow focus on a limited set of emotions has made this line of research lose sight of the great variety of emotional experiences that people have during their life-span and across different cultures, and of the relationship between these discrete, full-blown emotions and other affective states such as moods, preferences, and attitudes.

## The Problematic Evidence for the Existence of Basic Emotions

The Basic Emotion approach has also faced criticisms due to the lack of consistent empirical evidence for their claim that basic emotions are biologically hardwired affect programs. After decades of research, there is still no solid evidence for the existence of distinctive patterns associated with discrete emotions at the neural, physiological, and behavioral levels.

Regarding the evidence for dedicated brain systems associated with discrete emotions, the main conclusion drawn from recent reviews is that instead of discrete subsystems associated with each basic emotion, there are specific brain areas associated with specific behaviors (e.g., freezing, attacking, and smiling), which are *sometimes* present when emotions are elicited (Barrett, 2006a; Lindquist et al., 2012). Similarly, reviews of the evidence for distinct patterns of peripheral physiological activation have failed to find robust and consistent patterns distinguishing discrete emotion categories (Cacioppo et al., 2000; Kreibig, 2010; Stephens et al., 2010; Kragel and LaBar, 2013)^1^.

Regarding facial and vocal expressions of emotions, there is little and conflicting evidence for the claim that the patterns predicted by Basic Emotion theories such as Ekman and Friesen (1984) are present in spontaneous emotional expressions (Gosselin et al., 1995; Carroll and Russell, 1997; Camras et al., 2002; Scherer and Ellgring, 2007). Vocal expressions of emotions have been much less researched than facial expressions, and most of this research has been carried out using portrayed expressions as stimuli, so there is little data about the extent to which these posed expressions correspond to natural ones (Scherer, 2003).

The strongest piece of empirical support for the existence of Basic Emotions supported by biological affect programs is the finding that participants attribute the same emotional states to photographs of portrayed facial expressions above chance level (70% on average, according to Scherer et al., 2011). Nevertheless, this agreement level lessens when participants are asked to rate natural or milder expressions, when participants observe dynamic rather than static expressions, when researchers use open-ended questionnaires rather than lists of a few emotional adjectives, when participants rate expressions made by people from a culture different to their own; and importantly, when the stimuli consist of *vocal* expressions (Frank and Stennett, 2001; Elfenbein and Ambady, 2003; Kayyal and Russell, 2013; Nelson and Russell, 2013).

### Evidence for the Expression of Basic Emotions in Vocalizations

The most important argument for the claim that music expresses basic emotions is the existence of acoustic patterns in human vocalizations associated with the expression of discrete, basic emotions (Juslin and Laukka, 2003; Fritz et al., 2009; Koelsch, 2014). This claim is not clearly supported by empirical evidence so far. The most consistent finding of studies analyzing the acoustic qualities of emotional prosody is that these psychoacoustic cues correlate most clearly with differences in arousal. More specific acoustic patterns distinguishing variations in valence, or distinguishing discrete emotional states have been more difficult to identify (Bachorowski, 1999; Russell et al., 2003; Juslin and Scherer, 2005; Scherer et al., 2011). Scherer (2003), Juslin and Scherer (2005), and Scherer et al. (2011) have argued that this situation is due to the fact that most research has studied a limited number of acoustic cues, and has neglected arousal differences present within “emotion families” (e.g., the differences between “repressed” anger and “explosive” anger). In their joint paper, Juslin and Scherer (2005) go as far as proposing that affective states of a relatively weak intensity are probably only differentiated in terms of the arousal and valence dimensions (Laukka et al., 2005). This observation suggests that clear-cut psychoacoustic patterns could only be identified when emotional expressions are intense. In consequence, only when the vocal stimuli used in experimental research are posed and exaggerated (like the expressions traditionally used in facial emotional expression research), do researchers find psychoacoustic patterns associated with discrete emotions.

Juslin and Laukka (2003) carried out a review of 104 studies on vocal expression of emotion, and 41 studies on musical expression, and concluded that there are enough acoustic differences in emotional prosody to distinguish five basic emotions in vocalizations and music: Anger, Fear, Happiness, Sadness, and Love-Tenderness. However, an examination of this evidence for these patterns in emotional vocalizations shows that there are at least three reasons to be skeptical about this conclusion. Furthermore, we analyze their evidence for musical expressions of emotion in the next section.

First, the majority of the studies included in Juslin and Laukka’s (2003) review (87%) used portrayals by actors. This type of studies tells us how actors *think* emotions should be portrayed, rather than how they *actually* happen, -in other words, these conclusions lack ecological validity. Hence, their usefulness consists in informing us about people’s prototype or ideal expressions for hypothetical, full-blown emotional states (Motley and Camden, 1988; Fernández-Dols et al., 1997; Russell et al., 2003; Nelson and Russell, 2013). For instance, in an experiment by Banse and Scherer (1996), where they claim to have found acoustic patterns associated with discrete emotions, the authors used vocalizations portrayed by actors. Moreover, the patterns associated with discrete emotions were not identified in all the 1344 vocal samples obtained from the actors, but on a subset of 224 samples which were further analyzed because they were judged as “best acted.” And in a more recent study by Bänziger et al. (2014) where they also compared vocalizations portrayed by actors in French and German confirmed the finding that most psychoacoustic cues are associated with variations in arousal, and that there are small, or non-existent associations with variations in valence.

Second, most of the findings about associations between acoustic cues and discrete emotions indicate that most of these cues are the same for emotions that have the same level of activation (Juslin and Laukka, 2003, pp. 792–795). Sadness and Tenderness, the two emotions with low activation, correlate with slow speech rate, low intensity, low frequency energy, low mean fundamental frequency (*F*_0_), and downward contours. Whereas Anger, Fear, and Happiness, the emotions with high activation level, correlate with fast speech rate, high intensity, high voice intensity variability, high frequency energy, high mean fundamental frequency, low fundamental frequency variability, and upward contours.

Third, only two of the nine acoustic parameters summarized in Juslin and Laukka’s review distinguish emotions beyond their level of activation. But even there, the results do not point to robust and consistent differences. Juslin and Laukka conclude that *F*_0_ variability distinguishes Anger (high variability) from Fear (low variability). Nevertheless, there are almost as many studies that found that Fear is associated with high or medium *F*_0_ variability (*n* = 15) than the number of studies that found that it is associated with low variability (*n* = 17). In fact, if we exclude from this list a study that found that Fear is associated with both medium and low variability, and a study that found that this emotion is associated with both high and low variability, then the number of studies reporting low and high or medium variability is the same (*n* = 15), and the distinction between Anger and Fear in terms of *F*_0_ variability becomes less clear. The second acoustic cue that distinguishes emotional expressions beyond arousal in the review is the level of microstructural regularity of the voices (i.e., small variations in frequency and intensity). However, this finding is based only on 5 studies (out of 104), and they can be interpreted as distinguishing between positive and negative valenced emotions: Happiness and Tenderness are associated with microstructural regularity, whereas Anger, Fear, and Sadness are associated with microstructural irregularity.

In summary, in this section we have shown how, despite the predictions of Basic Emotion theories, there is little and inconsistent evidence for the existence of distinctive patterns associated with discrete emotions at the physiological, neural, and expressive behavior levels (i.e., facial expressions and speech prosody).

Before analyzing the evidence that music expresses basic emotions, it is important to clarify the scope of the criticism we have presented so far to the notion that emotions have associated facial and vocal expressions. Our claim is not that emotional episodes have absolutely no effects on facial and vocal behavior. It is very unlikely that emotions have no consequences on our facial behavior and on our speech prosody. Moreover, these effects should be more obvious in very intense emotional episodes, when the eliciting situation is so relevant and urgent that we feel overtaken by urges to attack, to hide away, to embrace someone, to be comforted, etc. Since all these action tendencies are associated with physiological changes in the autonomic nervous system (Frijda, 1986), they are probably also reflected in our faces and in the acoustic features of our voices (see Scherer, 1986, for specific hypotheses about the effects of appraisals on the physiology of vocalizations). In contrast, less intense emotional episodes and more diffuse affective states such as moods probably have less prominent physiological effects, and therefore, less clear effects on vocal and facial expressions.

Nevertheless, acknowledging that intense emotions involve changes in facial and vocal behaviors should not be taken as implying that every type of emotion is associated with a distinctive pattern of physiological and expressive behaviors. On the contrary, since every instance of anger, fear, joy, etc., is different, then there is no guarantee that the same action tendencies, physiological changes, and behaviors are present every time we experience these emotions. Consider the following examples: the experience of running into a bear in the woods, sitting in a doctor’s waiting room expecting a diagnosis of cancer, having to answer a difficult question in the context of a job interview, and listening to an eerie sound at midnight in a house where we assumed we were alone. Even though all of these experiences can be considered instances of “fear,” the different contexts in which they occur require us to respond in different ways, and therefore the pattern of physiological activation and the observable behavioral expressions would also be different in every case. Furthermore, since emotional responses are always tailored to the demands of the situation, the full pattern of expressive behaviors predicted by Basic Emotion theories are very seldom, if ever, observable in natural circumstances (Barrett, 2006a).

## Does Music Express Basic Emotions?

In this section we examine the claim that music expresses basic emotions. After all, even though the perception of emotion in music may not have its origin in discrete, biologically hardwired emotions, it is still possible that people perceive musically expressed emotions in categories that correspond to basic emotions.

As mentioned above, traditionally, researchers of musical expression of emotions have asked listeners to judge a set of discrete, basic emotions: Anger, Fear, Happiness, Sadness, and Love or tenderness (Eerola and Vuoskoski, 2013). Consequently, they have concluded that these emotions are expressed by music, and reliably recognized by listeners.

In our view, there are three problems with using these sources of evidence as the basis for determining which emotions music can express. First, asking listeners which emotions they think music expresses, inform us about people’s ideas about what emotions music expresses, not about their *actual experiences* of perceiving those emotions in music. Second, the evidence from experiments on perception of musical emotions involves a circular logic: most researchers assume *a priori* that music expresses a list of emotions, ask their participants to report their experience using the categories in that list, and conclude that in effect, music expresses the emotions they hypothesized. Third, and most importantly, the arguments for selecting which basic emotions music expresses should not only be empirical, but also, *theoretical*. To our knowledge, the advocates of the view that music expresses five basic emotions have not proposed a systematic conceptual account of *why* music should be able to express the set of basic emotions they propose. As a consequence, they have left two crucial questions unanswered.

The first question, is why these researchers have decided to include a category that appears in only a few Basic Emotion theories: *Love-Tenderness*. If the answer is simply that this category appears frequently in the lists of emotions that people more easily perceived in music, then why not include other common categories, such as “peacefulness”? Research into everyday experiences with music has found that two of the most frequently perceived affective states in music are calm or peacefulness (Lindström et al., 2003; Juslin and Laukka, 2004). Why then, not assume that ‘calm’ is a basic emotion?

The second question, is why out of all the emotions proposed within the Basic Emotions approach, advocates of the Basic Emotions view such as Juslin and colleagues have included only five categories (Happiness, Anger, Fear, Sadness, and Tenderness), in neglect of others categories such as Disgust, Contempt, Guilt, Shame, and Lust (c.f. Ortony and Turner, 1990 for different versions of Basic Emotions lists). Perhaps the answer is that the emotions most frequently included in music research are affective states that can be experienced without the need for an intentional object, whereas Disgust, Guilt, Shame, and Lust are always intentional states; that is, they are experienced directed to an object (e.g., every time we feel guilty, we feel guilty about something in particular). And since instrumental music is characterized by its inability to specify the object of the emotions it represents, then music’s ability to represent affective experiences is restricted to the expression of object-less affective states (Kivy, 1999; Davies, 2003; Cross, 2009). Although this might be a sensible argument, the Basic Emotion approach to musical expressivity could not adopt it, because it implies that music cannot express emotions but *moods*, which are the type of affective states that can be experienced without a clear intentional object. Hence, assuming this argument would ultimately contradict the central assumption of the Basic Emotions framework, that focuses on the phylogenetically inherited character of *emotions* (i.e., quick, object-directed, motivationally driving reactions), not of *moods* (i.e., slow, diffuse, cognitive-biasing states are experienced as directed toward the world in general, rather than toward a determinate object) (Frijda, 2008).

### Evidence From Developmental Studies

According to the Basic Emotions framework, expression and perception of basic emotions appear early in development (Harris, 1989; Izard, 1992; Juslin, 2013). If music expressivity is organized around basic emotions whose expression and perception appears early in ontogeny, then it follows that children’s perception of musical emotions should follow the same early developmental path.

The evidence from emotion development studies of in non-musical domains contradicts this assumption. Thus, until approximately age 3, children’s emotional vocabulary and perception is organized into broad categories representing the contrast between positive and negative experiences (Widen and Russell, 2008; Székely, 2013; Widen, 2013). Infants progressively incorporate more fine-grained categories such as sadness, anger, and fear when they reach the age of 4 or 5 (Bormann-Kischkel et al., 1990; Widen and Russell, 2008; Widen, 2013).

This process of development is not clearly paralleled in music. While some studies have found evidence for discrimination of valence expressed by music in children as young as 3 years, most studies have found that the ability to discriminate happy from sad musical excerpts above chance starts to emerge at some point around 4 or 5 years of age (Dolgin and Adelson, 1990; Adachi and Trehub, 1998; Mote, 2011; Stachó et al., 2013; Franco et al., 2016; but see Cunningham and Sterling, 1988; and Hunter et al., 2011, for two studies that found this ability only in later ages). Notably, the ability emerges around the same age when they develop the ability to entrain to musical rhythms, suggesting that tempo variations play a central role in the ability to distinguish these two expressions both in speech and music (Dalla Bella et al., 2001).

Several studies have found that young children tend to confuse angry and fear expressions (Terwogt and van Grinsven, 1991; Nawrot, 2003; Esposito and Serio, 2007). In fact, children’s ability to discriminate happy, sad, angry, and fearful expressions in music starts to appear around 6 to 8 years of age (Kastner Pinchot and Crowder, 1990; Kratus, 1993; Gerardi and Gerken, 1995; Dalla Bella et al., 2001; Nawrot, 2003), and their ability only reaches adult-like performance in emotion discrimination tasks of music much later: around age 11 (Hunter et al., 2011). The disagreements on the exact age where these abilities emerge may be attributed to differences in stimuli, procedure, and response formats used in each study (see Franco et al., 2016 for a review of these methods). However, beyond this variety, a developmental milestone that happens between 6 and 8 years of age explains the gradual development of discriminating several emotions expressed by music: the acquisition of sensitivity to mode, a musical cue associated with the expression of negative emotions in Western music (Gregory et al., 1996). Studies such as Adachi and Trehub (1998) and Dalla Bella et al. (2001) suggest that while younger children only rely on tempo variations to discriminate the emotional message expressed by music (a cue that is also present in vocalizations, and is therefore probably universal), older children and adults rely also on mode variations (and to some extent melodic contour, Gerardi and Gerken, 1995). Taken together, these findings suggest that early recognition of emotions in music relies on perceptual mechanisms that detect variations in arousal in vocalizations, such as tempo and loudness, but discrimination of discrete emotions depends on learning culture-specific cues such as mode. In sum, contrary to the predictions of Basic Emotion theory, perception of the whole set of basic emotions in music does not occur early in development, and it seems to depend on learning culture-specific cues such as specific associations between mode and mood.

### Evidence From Cross-Cultural Studies

If expression of emotions in music arouses from hardwired biological programs associated with the expression of basic emotions, then it follows that the striking findings about universal perception of facial expressions (Matsumoto et al., 2008; but see Nelson and Russell, 2013) should be paralleled in music too. In fact, music psychologists have embraced the central thesis of Elfenbein’s dialect theory of facial expressions of emotion (Elfenbein et al., 2007), and Thompson and Balkwill’s Cue-Redundancy Model of listeners’ perception of emotion in music (Balkwill and Thompson, 1999; Thompson and Balkwill, 2010). According to these two models, cross-cultural expression and communication of emotion (in facial expressions and music, respectively) is made possible by the existence of both universal and culture-specific cues. In consequence, the more universal cues are present in a piece of music, the more listeners unfamiliar with a piece of music from another culture can infer the same emotions expressed in that piece as enculturated listeners.

The evidence from cross-cultural studies on perception of musical emotions supports the general hypothesis that listeners are able to identify the intended emotional expression of music from a different culture (Thompson and Balkwill, 2010). What is less clear from this evidence, however, is that cross-cultural perception of musical emotions is organized around basic emotion categories. Several pioneering studies into this phenomenon had many methodological limitations, such as the use of *ad hoc* categories rather than standard emotional adjectives as dependent measures and participants have also been familiar with western music, making the comparability of results difficult (Gundlach, 1932, 1935; Morey, 1940; Deva and Virmani, 1975; Gregory and Varney, 1996). And while more recent have used standard emotional adjectives, they have usually explored the perception of only three categories: Joy, Sadness, and Anger (e.g., Balkwill et al., 2004; Fritz et al., 2009), and in consequence their results are open to an alternative, dimensional explanation. Namely, the fact that these emotions correspond to different combinations of activation and valence levels (Russell and Barrett, 1999), makes it possible that the participants’ accurate responses were due to their ability to distinguish the difference between an energetic and positive emotion and a subdued and negative one, rather than between Joy and Sadness, for example. In other words, the results from these studies make it impossible to discard the hypothesis that the participants’ perception is organized around general affective dimensions rather than around discrete categories. Thus, participants in these experiments tended to choose the “correct” emotional adjective, because they detected the levels of arousal and valence specified by the music, and they used contextual cues to figure out the discrete emotional category that better fitted with those arousal and valence levels. In the context of these experiments, these contextual cues might have been provided by the use of close-ended response formats, which bias the listener’s perceptual experience. (We return to this point in section “The Role of Contexts in the Perception of Emotional Expressions”).

A recent experiment by Laukka et al. (2013) sought to overcome these and other limitations of past research, such as the tendency to use Western music as the stimuli that listeners have to judge. In this experiment, in addition to using Western classical music excerpts, the researchers asked Swedish, Indian, and Japanese musicians to create music to express 11 different emotions and affective states (anger, fear, happiness, affection, humor, longing, peacefulness, sadness, solemnity, spirituality, and neutral), which were later judged by listeners from the same three cultures. The researchers also analyzed the extent to which musicians and listeners use the same acoustic cues to encode and decode the intended affective expressions. The results from the experiment largely support the researchers’ predictions. The listeners were better at identifying basic emotions (anger, fear, happiness, and sadness) than non-basic ones (e.g., solemnity, humor, and longing). And even though they were equally good at recognizing the emotional expression intended by Western classical music excerpts, they were better able to identify the intended emotions in music from their own culture than from an unfamiliar one. Although the results are encouraging in several ways, the conclusions need to be qualified by the following considerations.

First, the pattern of confusion exhibited by participants, (i.e., the distribution of occasions when they misattributed the intended expression in the music) was consistent with the view that participants were sensitive to the activity and valence dimensions of music.

Second, the acoustic cues associated with the expression and perception of discrete emotions that have the same level of activity and valence show a large number of coincidences. These coincidences, however, are more marked across those cues that are common to vocalizations and music (such as intensity, timbre, and pitch height), than across those cues that can only be found in music (such as modality, tonal, and rhythmic stability). This suggests that even though the listeners’ sensibility to the first type of cues may have helped them identify the level of arousal and valence expressed by the music, the musically specific cues were critical for the listeners’ ability to differentiate emotions with similar levels on those dimensions.

Third, some emotions considered “basic” and therefore universal, were not correctly identified above chance levels, sometimes even by members of the same culture. For example, Happiness was only correctly identified in Western classical music and Swedish folk music; Sadness in Japanese music was not recognized by most Japanese listeners, and Sadness in Swedish music was not recognized by most Indian listeners. Affection, the emotion category most closely related to the “tenderness/love” category proposed as a basic emotion by Juslin and colleagues, was not correctly identified in any of the non-Western musical styles (the only exception was Indian music, were it was identified only by Indian listeners). This finding that several basic emotions were not identified even within listeners of the same culture contrasts starkly with the high accuracy levels exhibited by participants of experiments on cross-cultural perception of facial and vocal expressions (c.f. Scherer et al., 2011).

In conclusion, the evidence from cross-cultural studies of expression and perception of musical emotions supports the hypothesis that expression of emotions in music is grounded on acoustic cues shared with vocalizations, and that these cues can at least signal variations in levels of arousal and valence. The evidence for universal musical expressions associated with discrete emotions is only partial, and it suggests that this fine-grained differentiation might depend more on cues that are present in music, but not in vocalizations. Clearly, further studies using methods such as the one implemented by Laukka et al. (2013) are needed to advance in understanding this phenomenon.

### Evidence for Shared Psychoacoustic Cues in Speech Prosody and Western Music

The strongest piece of evidence for the expression of basic emotions in music is the already mentioned review of 145 studies into emotional expression vocalizations and music carried out by Juslin and Laukka (2003). This evidence, however, is not completely unambiguous. Although the results of most studies support the prediction that acoustic parameters associated with the expression of emotion in vocalizations show the same patterns of association in music, the evidence for the claim that the acoustic parameters that *discriminate specific emotions* in music are the same for vocalizations is less clear.

The meta-analysis paper by Juslin and Laukka (2003) shows that most of the acoustic parameters associated with specific emotions in music do not present the same pattern in vocalizations. First, in music, Fear and Anger are distinguished by sound level (high in Anger, low in Fear), but this distinction is not paralleled in vocalizations, where both emotions are associated with high sound level. Second, in music, Happiness is associated with little sound level variability, whereas in vocalizations, it is associated with high variability. And third, in music, timbres characterized by abundant presence of high-frequencies are associated with Anger, timbres with moderate number of high-frequencies are associated with Happiness, and timbres with few high-frequencies with Fear. In vocalizations, all emotions with high levels of activation (Anger, Fear, and Happiness) are associated with abundant presence of high frequencies.

The evidence from Juslin and Laukka’s (2003) review can be complemented by more recently published experiments into shared psychoacoustic cues to the expression of emotions in music and speech (Illie and Thompson, 2006; Curtis and Bharucha, 2010; Scherer et al., 2011, 2013; Bowling et al., 2012; Weninger et al., 2013); and by experiments on musical parameters associated with expression of emotion (Costa et al., 2004; Schubert, 2004; Juslin and Lindström, 2010; Eerola et al., 2013; Quinto et al., 2014). As can be seen in **Table 1**, in general terms this more recent evidence coincides with the results of Juslin and Laukka’s (2003) review.

**Table 1:** Summary of findings of psychoacoustic parameters associated with emotional expression in vocalizations and music published after Juslin and Laukka’s (2003) review.

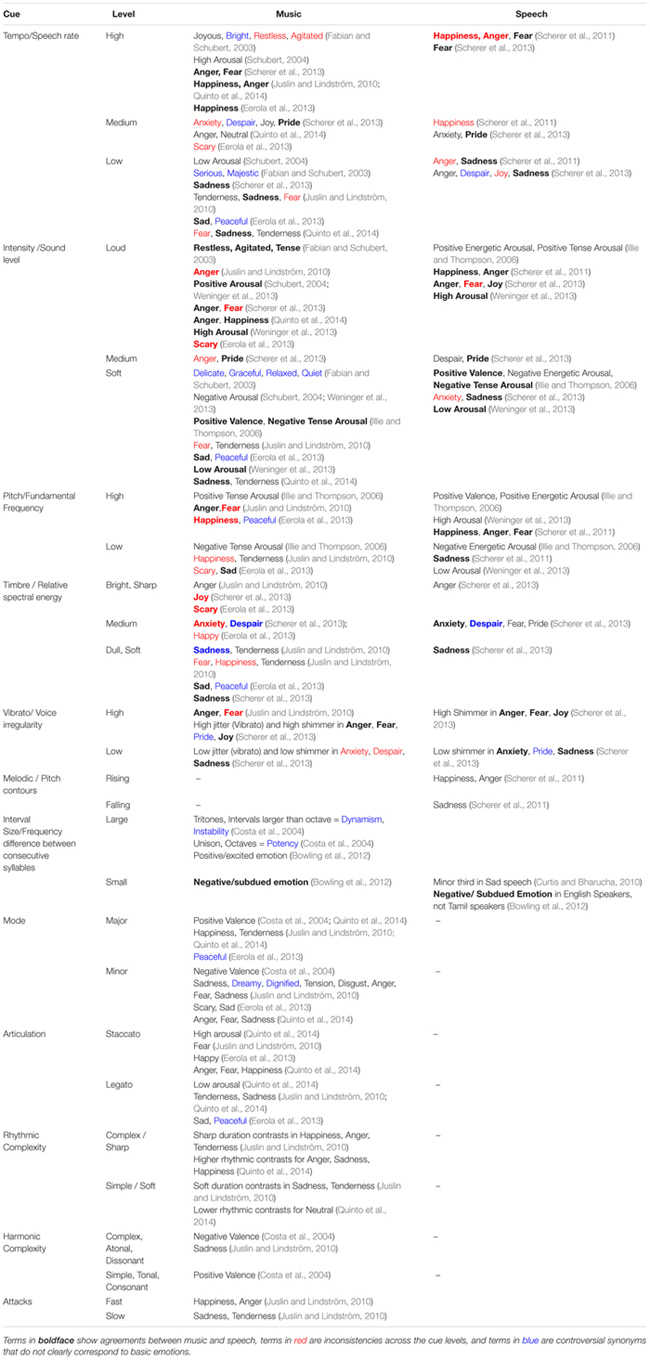

To further explore the parsimony of these cue-emotion combinations using either basic emotions or emotion dimensions, we subjected reported cue-affect combinations from the existing studies to correspondence analysis (CA), which attempts to represent them with an optimal number of eigenvectors. We took all studies reporting acoustic or musical cues contributing to emotions or quadrants in the affective circumplex in music (53 studies) and speech (82 studies) published after Juslin and Laukka’s (2003) review. We focused on 15 cues (intensity, tempo, frequency, timbre, jitter, mode, articulation, rhythmic complexity, harmonic complexity, attacks, intensity, variability, jitter, contour, microstructural regularity) and 5 basic emotions (anger, fear, happiness, love-tenderness, and sadness) and 4 quadrants of affect dimensions (high arousal positive valence, high arousal negative valence, low arousal positive valence and low arousal and negative valence). Since the basic emotion terminology varies across the studies, we reduced the variant terms into 5 basic emotions using Juslin and Laukka’s (2003) classification of emotion terms. This amounted to 1243 cue-emotion pairs in speech and music. The CA determined the optimal decomposition of cues to affect categories in basic emotions and dimensions in the two domains (music and speech). **Table 2** displays the decomposition summary and the variance explained.

**Table 2:** Correspondence Analysis: Variance explained across Domains and two emotion mappings (Basic Emotions and Quadrants of the Two Dimensions).

	Music		Speech	
	Basic emotions	Dimensions	Basic emotions	Dimensions
Dim 1.	34.5%	46.2%	68.0%	72.2%
Dim 2.	26.3%	29.6%	20.2%	21.4%
Dim 3.	22.2%	24.2%	6.6%	6.4%
Dim 4.	17.0%		5.2%	
*X*^2^(CI)	29.2 (27.5–31.4)	24.6 (22.7–26.5)	18.3 (17.1–19.6)	16.6 (15.4–17.7)

For Basic Emotions, the analysis offers consistently higher number of necessary eigenvectors (4 vs. 3 dimensions) than for the quadrants representing the affect dimensions when representing the full contingency table of cues and emotion terms. This suggests that the quadrants of the dimensional representation capture the configuration in a more parsimonious fashion than the Basic Emotions. Similarly, the first two dimensions capture more variance in dimensional mapping scheme in comparison with the Basic Emotions (75.8 and 93.6% for dimensions and 60.8 and 88.2% for Basic Emotions in music and speech, respectively). Also, the chi-square distances are consistently smaller for the decomposition of dimensions to cues in comparison to Basic Emotions (with non-overlapping bootstrapped confidence intervals). Also, the cue-emotion combinations are somewhat simpler and more redundant in speech in comparison to music, which is similar to past results in emotion recognition in speech and music (Johnstone and Scherer, 2000; Juslin and Laukka, 2003). In sum, the results from the CA corroborate that the mapping between acoustic cues in speech and music fit a dimensional model (according to which, music communicates arousal and valence), better than a Basic Emotions one (according to which, music communicates a set of discrete emotions).

Taken together, the evidence from cross-cultural and developmental studies, and from research into the expression of emotion in vocalizations and music leads to the following conclusions:

(1) There is a great number of coincidences between acoustic patterns in speech prosody and in music. These coincidences are consistent with the view that the perception of emotional expressions in music and in vocalizations depends, at least partly, in shared psychological and neural mechanisms (Escoffier et al., 2012).
(2) Just as found in research into emotional vocalizations in general, most of the parallels between psychoacoustic cues to emotional expression in speech prosody and music can be mapped onto different levels of arousal^2^.
(3) If we limit the analysis to the cues that are both present in prosody and music, it is difficult to find consistent and unambiguous patterns that can be mapped onto variations in valence and/or discrete emotions. At the same time, the more we include cues present exclusively in music (such as modality, and harmonic and rhythmic complexity), the more we find distinct associations between configurations of acoustic cues and the expression of specific emotions.^3^
(4) Conversely, as predicted by Juslin’s (1997, 2001, 2003) model, most studies have found that the more cues are present, the more participants can successfully recognize discrete emotions, confirming the above-mentioned facilitating effect that the use of exaggerated prototypes has in the discrimination of emotions by observers (Frank and Stennett, 2001; Nelson and Russell, 2013). It is unclear, however, the extent to which the music that people choose to listen in their everyday lives, (as opposed to music used in experimental studies) makes use of these stereotyped acoustic configurations. There is evidence for example, that valence is expressed in different ways across musical genres (Eerola, 2011).
(5) The fewer music-specific cues are present, the more people who are not familiarized with them have difficulties identifying the intended expressed emotion in music (i.e., children, and listeners from non-Western cultures). Nevertheless, the analyses of the pattern of misattribution made by participants in the experiments reveals that listeners are sensitive to the levels of activity and valence expressed by music.
(6) The results from some of the studies published after Juslin and Laukka’s (2003) review contradict each other’s findings, and Juslin and Laukka’s conclusions. These inconsistencies can be attributed to several reasons. First, there are important differences in procedures, materials, and measurement scales across studies. In particular, discrepancies in the way emotions are labeled can lead to different results. For instance, it is not the same to ask musicians to produce music that sounds angry than to ask them to produce music that sounds frustrated, irritated, or furious; and likewise, these adjectives are not necessarily equivalent from a listener’s point of view. Second, it is possible that some of the inconsistencies in the psychoacoustic cues associated with the expression of emotions are due to the presence of interactions between several cues (but see Eerola et al., 2013). Thirdly, a most parsimonious explanation is that often the underlying dimensions would explain the same patterns, and therefore the success of discriminating basic emotion categories cannot be taken at a face value of providing positive evidence for these.

## From Affective Dimensions to Categorical Perception of Emotions

As mentioned above, the best support for the existence of Basic Emotions is the finding that when participants are asked to judge the emotion communicated by a portrayed facial, vocal or musical expression, they agree in the correct answer above chance level^4^ (Scherer et al., 2011). This finding, however, entails a paradox: people’s perception of these stimuli is clearly organized into categories, and they tend to agree as to which categories correspond to every stimulus they judge. However, these categories do not seem to be present *in the stimuli* whether it be facial expressions, vocalizations, or musical materials. As we have argued, there is little evidence that the predicted facial vocal patterns occur in natural circumstances; the evidence for expressive patterns associated with discrete emotions is elusive (particularly in vocalizations); and the acoustic cues of emotional expression shared by vocalizations and music are more clearly related to arousal than to discrete emotions. In other words, whereas *objective* measures of emotional expression have failed to find distinct categories, people’s *subjective* perception of emotion is categorical (Barrett, 2006b). As we show in this section, this paradox can be resolved by considering the way cultural and perceptual categories are constructed, and the crucial role that context has in the perception of emotional expressions.

### Discrete Emotional Categories Are in the Eye (and the Ear) of the Beholder

The first argument that helps dissolve the paradox can be found (surprisingly, given our preceding critique) in a passage of a paper by Juslin (2013). When confronted with the above-mentioned inconsistency, Juslin concedes that discrete categories exist in people’s minds, not in the materials (facial expressions, voices, or music):

“It’s clear that the acoustic patterns obtained do not always neatly correspond to categories. But to look for discrete categories in the acoustic data is to look at the wrong place altogether. Categorical perception is *a creation of the mind*, it’s *not in the physical stimulus*” (Juslin, 2013, p. 5 italics added).

The importance of this observation is paramount, because it suggests that the findings about universal perceptions of emotions are not due to emotions having a common, discrete biological substrate, but to the existence of common emotion *concepts* that organize people’s perception of emotions. Indeed, the existence of a limited, universal set of emotion concepts in people’s perceptual systems and languages need not arise from a set of biologically predetermined discrete emotions; it can simply occur because all humans across cultures face the same relevant events (e.g., facing a threat, losing something valued, confronting goal-obstructing situations, discovering outcomes that are better than expected, etc.). If all human beings face the same type of goal-relevant situations, and they evaluate them in similar ways, then it follows that all cultures must create similar conceptual and linguistic categories to denote them (Scherer, 1994; Frijda, 2008)

Nevertheless, the existence of these common conceptual and linguistic categories does not completely dissolve the paradox. The existence of cross-culturally shared categories does not explain why, when presented with exaggerated, posed expressions, most participants attribute the same emotional category to the same stimuli, and why they still tend to select the same category when they judge facial, vocal or musical stimuli portrayed by people from other cultures. Hence, the second argument that dissolves the perceptual paradox has to be found in an examination of the way people use and construct mental prototypes.

Research into the construction of mental categories has shown that people construct ideal representations to categorize similar objects, even when they have never seen an object containing all the features of the ideal representation. Particularly in the domain of face recognition, a number of studies have demonstrated that when participants are presented with a number of similar faces, they implicitly build prototypes “averaging” their features, and that these prototypes are so strong that they create false memories of having seen them before (Solso and McCarthy, 1981; Bruce et al., 1991; Cabeza et al., 1999; De Fockert and Wolfenstein, 2009). Similarly, another line of research has also shown that even when researchers present participants with large numbers of stimuli that gradually vary along a continuum, they perceive them as separated by boundaries that divide them into discrete categories (Young et al., 1997; Laukka, 2005; Brosch et al., 2010).

Taken together, the findings from these two lines of research help understand how, although the exaggerated facial and vocal stimuli used in emotion expression research actually occur rarely in spontaneous interactions, people construct ideal representations or prototypes, which influence perception of emotionally expressive stimuli in a top-down manner, creating artificial discrete categories (Brosch et al., 2010).

The same process of prototype construction which leads to categorical perception probably occurs in perception of emotional expressions in music too. Although the exaggerated emotional expressions used in experimental research may rarely be found in music that people listen to in everyday circumstances, they are easily identified as belonging to basic emotion categories because people’s perception of emotional expressions is based on categories that use the average prototype as a guide for classification. Additionally, it is also likely that in the case of Western listeners, these mental prototypes are also derived from their exposure to culturally shared images and symbols such as the classic Greek images for comedy and tragedy, the facial and vocal expressions of cartoons, and the associations between visual narratives and music soundtracks.

### The Role of Contexts in the Perception of Emotional Expressions

Implicit to the Basic Emotion approach is the assumption that emotional meanings are inherent to facial, vocal, and musical expressions, and therefore they can be readily decoded by perceivers, independently of the situation where the expression is displayed. This assumption is based on an evolutionary argument, according to which, it is adaptive for animals to communicate discrete emotional categories using fixed expressive patterns, which can be recognized by an observer in any circumstance (Ekman, 1992; Juslin and Laukka, 2003). This assumption in turn, has inspired hundreds of studies where researchers have attempted to identify emotional meanings in facial, vocal, bodily, and musical expressions that can be identified by any observer, in any situation.

The problem with the evolutionary argument put forward by the Basic Emotions tradition is that it assumes that expressive gestures and vocalizations always originate in an underlying emotional state, and that they are always perceived as communicating emotions by observers, as if humans and animals ever expressed and perceived emotions in context-free situations. Ethologists, -researchers of animal communication, have shown how evolution has favored flexibility over rigidness, and the communication of social intentions over emotional states, even in non-human primates (Parr et al., 2005; Demuru et al., 2015; Fröhlich et al., 2016). This alternative view proposes that the gestures displayed by an animal in a given circumstance depend on the demands of the situation, and that it is more advantageous for an animal to display gestures to communicate intentions and to influence other animals, rather than to show its emotional state (Fridlund, 1994; Bachorowski, 1999; Rendall et al., 2009). For example, it is more advantageous for a primate to display an expression of anger when it wants to intimidate a rival (thus preventing the confrontation from happening), than when it has the intention of attacking and overcoming its rival immediately (Fridlund, 1994). Similarly, studies with human participants have shown how emotional expressions vary according to the characteristics of the situation, and communicate different intentions accordingly. For instance, people do not necessarily smile more when they experience positive results on their own, but they do smile more when they communicate those positive results to other people (Kraut and Johnston, 1979; Ruiz-Belda et al., 2003). Also, different types of smiles are associated with different social intentions, and are perceived accordingly. For example, embarrassment smiles seem to have the function of appeasing the negative judgment of observers, whereas enjoyment smiles have the function of increasing closeness with others (Niedenthal et al., 2010).

That the interpretation of emotional expressions is flexible and tailored to the situation where they occur is also evident in the way observers perceive different meanings in facial expressions and vocalizations according to contextual information. Several experiments on perception of emotional expressions have demonstrated this effect (see Barrett et al., 2011 for a review of the evidence). For example, Carroll and Russell (1996) showed how even exaggerated portrayals of emotions can be perceived as expressing different emotions, or even non-emotional states when they are associated with different contexts. For instance, when participants observed a face showing the prototypical anger expression with frown eyebrows and bare teeth, they perceived it alternatively as expressive of anger, fear, or physical exertion, depending on the narrative they read about the situation that led the person to make that facial expression.

A defender of the Basic Emotion approach could reply to this argument by saying that in a psychological experiment, the participants who judge the portrayed stimuli encounter them in a context-free situation. Yet this argument can be challenged by considering that in these experiments, the context is provided by the list of emotional adjectives that the participants have to choose from to make their judgment. These lists effectively restrict the number and type of inferences that participants can make about the psychological state of the person portraying the expression, and therefore bias their perception of it (Russell, 1994; Frank and Stennett, 2001). Research has shown that when instead of close-ended questionnaires, investigators use open answers, or tasks asking participants to match two faces expressing the same emotion, agreement among participants diminishes dramatically (Nelson and Russell, 2013).

In the music domain, the biasing effect that response formats have on perception has been demonstrated in studies where researchers ask participants to rate music in non-emotional terms, such as sharpness, weight, smoothness, moisture, and temperature (Eitan and Rothschild, 2011); movement (Eitan and Granot, 2006; Sievers et al., 2013); spatial height, mass, strength, and brightness (Eitan and Timmers, 2010); and people’s traits (Watt and Ash, 1998). In all of these experiments, researchers have observed high levels of agreement in participants’ ratings, suggesting that musical meanings, just like facial and vocal expressions, are flexible, not inherent to the musical materials, and not restricted to a few standard emotional categories.

In sum, the consideration of the role that contexts play biasing the perception of emotional expressions is a third argument that resolves the paradox: people tend to agree on the emotions expressed by facial gestures, vocalizations, and music, because they find significant cues in the situation, and the response format that they are asked to use to make their decision.

### A Constructionist Account of the Perception of Discrete Emotions in Music

In this final section, we draw from constructionist theories of emotion (Russell, 2003; Barrett, 2006a; Barrett et al., 2007), constructionist theories of musical meaning (Cook, 2001; DeNora, 2003), and ecological theories of music perception (Dibben, 2001; Clarke, 2005) to propose an alternative view of the phenomenon of expression and perception of musical emotions. What these theories have in common is the assumption that emotional or musical meanings are not inherent in expressive behaviors and musical sounds, but emerge from the interaction of the materials (i.e., the configuration of the facial expressions, the acoustic qualities of the voice, or the structure of the musical work), the knowledge and goals of the observer, and the characteristics of the situation where the expressive behavior or musical work occurs.

According to Barrett’s Conceptual Act Theory (Barrett, 2006a,b, 2011), emotional experiences occur much in the same way as the perception of colors. Although shades of colors consist of continuum wavelengths, we perceive them categorically, because in the act of perceiving a color of an object we quickly combine top-down information (such as knowledge of linguistic labels for colors and typical objects associated with them) with bottom-up sensorial information, creating the experience of seeing discrete colors (Barrett, 2006b, p. 27). Analogously, for the Conceptual Act Theory, the experience of having an emotion and the experience of perceiving an emotion in another person occur when top-down knowledge from past emotional experiences is quickly combined with information about the present situation, and sensory information from our own body, or from the other person’s behavior. In the case of experiencing emotions in oneself, the most important source of sensorial information consists of fluctuations of *core affect*, an underlying affective tone experienced as variations in valence (feelings of pleasantness) and arousal (feelings of activation) (Russell and Barrett, 1999). In the case of perceiving emotions in another individual, the sensorial information consists in the behaviors of the other person, which at the very least, signal that person’s core affect (i.e., how activated and pleasant he or she is feeling) (Carroll and Russell, 1996). Barrett calls the process of categorization of core affect a *conceptual act*, in order to emphasize the immediacy of the process, and its dependence on the existence of previously acquired knowledge, (including implicit linguistic knowledge of emotion categories). Thus, for Barrett emotional experiences are context-dependent episodes that emerge from the combination of more basic psychological and physiological processes, and are not determined by the triggering of biologically pre-determined affect programs associated with prototypical stimuli or expressions (as assumed by Basic Emotion theories).

How can this theoretical framework be adapted to the case of the perception of emotions in music? Our claim is that, although there is enough basis to conclude that expression of emotions in music is ultimately founded on an overlap with the mechanisms of emotional expression in vocalizations, when we strip music from culture-specific cues, and we focus exclusively on those acoustic parameters present both in emotional prosody and music, we are left with an essentially ambiguous material that can only specify variations of arousal, -and to a lesser extent, of valence (i.e., core affect). However, musical sounds afford the perception of specific, discrete meanings (including emotional ones) when the listener’s mind combines top-down knowledge from past musical experiences, information about his or her current affective state, and cues about the meaning of the event where the music is playing.

Consistent with this constructionist approach, we claim that perceiving emotions in music consists of an active process of meaning construction, where the ambiguous affective information provided by the music acoustic cues becomes differentiated and categorized into discrete meanings in a conceptual act. This ambiguous information becomes differentiated into discrete percepts thanks to associative mechanisms that integrate a variety of sources of information effortlessly and automatically. Some of these sources of information have their origin in implicit psychological processes such as the process of prototype construction described above, and the use of linguistic labels that organize emotional experiences into discrete categories (Lindquist, 2009). Other sources of information are originated in cultural conventions such as the association between mode and musical valence, and in personal associations, such as the use of musical genres for mood-regulation strategies (e.g., listening to a piece of classical music to experience relaxation). Finally, other sources of information are context-specific, such as the listener’s current mood and goals, the presence of lyrics with emotional content, the presentation of visual narratives presented along the music (e.g., in a movie), the observation of gestures made by the musicians, and the listener’s sensitivity to the cultural meaning of the situation where the music takes place (e.g., a funeral, a mass, a graduation ceremony, etc.).

It is important to note that this proposal does not amount to saying that musical meanings are completely free, idiosyncratic, and as variable as the contexts in which they occur. On the contrary, drawing from the ecological perspective to music perception mentioned above, our claim is that musical structures *afford* certain meanings to be privileged over alternative ones (Dibben, 2001; Clarke, 2005). Moreover, since musical perception of emotions is built on our ability to perceive variations of arousal and valence in speech, this shared code biases the musical meanings that people attribute to music, making them coherent with the level of activity and pleasantness expressed by the musical structures. For instance, it is unlikely that listeners perceive a loud, dissonant, and fast piece of music as expressive of tenderness and that they use it as a lullaby, because the objective qualities of the music are incompatible with relaxed bodily states and cultural notions of motherly love.

At this point, we deem it necessary to point to two important areas of coincidence and difference between our proposal and the Basic Emotions approach to music expressivity, proposed by Juslin and colleagues, and with the theory of dynamic communication of core affect proposed by Flaig and Large (2014).

In the first place, the constructionist approach here proposed *complements*, rather than replaces the lens model proposed by Juslin (1997, 2003) and Juslin and Lindström (2010). The lens model, with its emphasis on the process of encoding and decoding of psychoacoustic cues, finds it hard to explain how it is possible that people can identify the correct emotional expression when there are few cues present in the musical material, and/or when they are not perceived by listeners. From our perspective, this paradox is resolved by considering the role of contexts and of musical and emotional knowledge in the construction of musical meanings. Thus, contextual clues, and the sources of information described above can lead to the perception of emotional and non-emotional meanings in the music even when the musical materials do not correspond to the prototypical stimuli used in most experimental research.

Second, the fact that music can express non-basic emotions and other affective states is to some extent acknowledged in Juslin’s theory of musical expressivity (Juslin, 2013). In his model, three layers of coding explain music’s ability to represent basic emotions, and non-basic emotions such as hope and solemnity: an iconic layer that communicates basic emotions, an intrinsic layer that communicates fluctuations of tension, and an associative layer that communicates “arbitrary” associations (Juslin, 2013, p. 4). In our view, it is unnecessary to propose the existence of these layers. We find it more parsimonious to dispose of the idea that the iconic level denotes discrete basic emotions, and to assume that music communicates fluctuations of affect which can be mapped onto many possible meanings via associative mechanisms.

Third, the constructionist framework we propose has many points of coincidence with the theory of dynamic music communication of core affect proposed by Flaig and Large (2014), according to which, music communicates primarily core affect thanks to processes of non-linear resonance between musical structures and patterns of neural oscillation. However, whereas the focus of their theory is on the neural mechanisms responsible for the perception of the affect specified by music, the focus of ours is on the psychological processes that transform those fluctuations of core affect into the experience of perceiving a discrete emotion expressed by music. In this sense, our theory complements Flaig and Large’s (2014) one, by specifying the processes of categorization that make listeners experience a variety of emergent emotional percepts according to the characteristics of the personal, situational, and cultural context where the music takes place.

## Conclusion

In this article we argued that despite the widespread assumption that musical expressivity is organized around a limited set of discrete, biologically pre-determined basic emotions, there are serious theoretical and empirical arguments that contradict this claim. We demonstrated that although there is evidence for the claim that the expression and perception of musical emotions arises from mechanisms that are shared with the expression and perception of speech prosody, this common biological ground is not organized around discrete categories. We also showed how the perceptual paradox, (consisting of the inconsistency of findings from objective and subjective measures of emotional expression), can be resolved by considering that the categorical perception of emotional expressions emerges from: (a) the existence of common linguistic categories, (b) the construction of ideal representations which create the illusion of the existence of prototypical expressions in natural situations; and (c) the disambiguating effect that contextual information has in the perception of emotional expressions. Thus, we submit that there is no need to invoke the existence of hardwired basic emotions to explain how people perceive categories in vocalizations and in music. Instead, we submit that this phenomenon can be better accounted for by adopting a constructionist approach to emotions. In this approach, the acoustical cues present in music can be mapped onto variations of core affect (i.e., activation and valence), which become discrete percepts thanks to the onset of quick associative mechanisms that integrate information from past knowledge, contextual information, and the listener’s current psychological state.

The proposal that people’s perception of meanings in music is flexible and varies according to different listening contexts has several implications for research into musical emotions. First, this perceptual flexibility suggests that finding that listeners *can* identify discrete emotions in music, does not suggest that people usually engage with music with the primary *objective of decoding the emotions that it expresses*. Moreover, people’s ability to perceive discrete emotions in music does not suggest that when people perceive emotions expressed by music, they experience them as discrete categories, or that the categories they perceive correspond to the discrete emotional adjectives that experimental research has investigated (Clarke, 2014). Hence, adopting this constructionist approach to musical emotions implies a shift in the focus of research from identifying associations between musical structures and emotion percepts, to identifying the *conditions* under which people perceive emotional meanings in music, and the conditions under which they perceive non-emotional ones.

Second, studying these sources of variation in people’s perception of emotions in music, involves studying how these meanings are constructed in everyday life contexts. On most occasions, people listen to music embedded in “extra-musical” elements such as lyrics, videos, photographs, social events, the presence of other listeners, etc. Given that all this contextual information has pronounced impact on the listeners’ emotional experiences with music (Eerola et al., 2015) studies should start mapping the influence of these factors in people’s perceived meanings in a systematic manner.

Third, we have argued that the affective information that music “by itself” can provide consists of variations of core affect: arousal and valence. However, it is conceivable that these two dimensions do not exhaust all the affective information that musical materials afford, and that listeners are sensitive to variations of energy and tension (Schimmack and Grob, 2000) or of power (Fontaine et al., 2007). Future studies should attempt to determine which dimensions, besides arousal and valence, underlie musical expression of emotions, and the contextual conditions under which these dimensions become more salient and differentiated.

Fourth, several researchers have proposed that one mechanism that leads to the *induction* of emotions by music (i.e., the experience that music *changes* our emotional state) is *emotional contagion*, whence we perceive that a piece of music expresses a particular emotion, and we feel that the same emotion is aroused in ourselves (Juslin and Västfjäll, 2008; Davies, 2010; Schubert, 2013). According to the BRECVEMAC theory proposed by Juslin and Västfjäll (2008) and Juslin et al. (2010, 2016), musical emotional contagion occurs because the perception of basic emotions in music triggers processes of internal mimicry in the listener, which in turn lead to an induction of the same emotion (Juslin and Västfjäll, 2008, p. 565). Adopting the constructionist approach to musical expressivity implies that even on those occasions when we observe a correspondence between perceived and induced emotion, we should not assume that the perceived basic emotion was the only, nor the main factor driving the listener’s emotional experience. Given that contextual, personal and cultural factors produce variations in experiences of perceiving emotions expressed by music, it is likely that they also influence the quality of the emotion aroused in the listener.

Fifth, the constructionist approach here proposed also has methodological implications. Despite the knowledge that decades of research into the association between musical structures and perception of emotion have provided, we will not advance our understanding of this phenomenon by continuing to use experimental designs where stimuli have stereotyped musical configurations, and response formats consists of close-ended lists of basic emotion adjectives. In our view, the way out of this circular logic is to start using more ambiguous musical stimuli, open-ended response formats, qualitative data about the listener’s perspective, manipulations of contextual information, and priming of cultural knowledge. Only by expanding the scope of research in this way can we learn how factors in the musical materials, the context (e.g., lyrics, visual narratives, program notes), and the listener’s knowledge interact in the process of construction of perception and meaning-making. Given that conceptual acts usually occur quickly, automatically and non-consciously, self-report measures should be complemented with physiological and implicit ones that do not depend on participants’ introspection. Moreover, the emphasis that this theoretical approach makes on the variety and flexibility of people’s emotional experiences with music, implies that variation in listener’s reports should not be discarded as errors of measurement, but regarded as informative data that needs to be incorporated and explained.

Finally, we submit that adopting the constructionist approach to perception of emotions in music can further our understanding the variety of emotional meanings are constructed in contexts such as musical videos, film music, advertisements, and music therapy. Already the applied psychology of music has taken this road by starting to focus on the contextual uses of music; music and well-being studies consider emotions as something which are essentially active regulation of one’s mood in a particular context (Saarikallio, 2011). Similarly, Music Information Retrieval (MIR) has taken the contextualized approach seriously when developing better recommendation services by incorporating situational information and personal information to aid mood discovery (Yang and Liu, 2013). In the same sense, this theoretical approach is better suited than Basic Emotion approaches for building much needed bridges between music psychology and other disciplines interested in understanding people’s affective experiences with music such as ethnomusicology, historical musicology, popular music studies, sociology of music, and music therapy.

## Author Contributions

JC-G wrote the main text of the article and contributed to the discussion of the theoretical proposal. TE performed the correspondence analysis of the association between acoustic cues and expression of emotions in vocalizations and music, wrote several sections of the paper, and contributed to the discussion of the theoretical proposal.

## Conflict of Interest Statement

The authors declare that the research was conducted in the absence of any commercial or financial relationships that could be construed as a potential conflict of interest.

## Footnotes

1 Two of these studies claim to have found distinctive patterns of autonomic activation associated with basic emotions. However, although these studies used similar pattern classification methods and stimuli, they did not replicate each other in the patterns they report (Stephens et al., 2010; Kragel and LaBar, 2013)

2 A notable exception is musical expression of Fear, which does not share some of the basic psychoacoustic cues found in emotional speech. However, an analysis of the features of the “fearful” stimuli in most experiments suggests that in this category, the distinction between expressed and induced emotions has been blurred. In other words, it is unclear whether music is supposed to portray the experience of *someone scared*, or to *frighten* the listener (Vieillard et al., 2008).

3 An intriguing exception is a study by Curtis and Bharucha (2010) who found that expression of sadness in speech vocalizations by English speakers was associated with pitch variations equivalent to the minor third interval in music. This result was replicated by Bowling et al. (2012) with a different sample of English speakers, but not with a sample of Tamil speakers. Hence, further replications with larger samples of languages are necessary before accepting this hypothesis.

4 Admittedly, this level of decoding accuracy is lower for vocal expressions (around 59%) than for facial expressions (around 77%).
